# QTL mapping in diploid potato by using selfed progenies of the cross *S. tuberosum* × *S. chacoense*

**DOI:** 10.1007/s10681-018-2191-6

**Published:** 2018-06-26

**Authors:** D. Meijer, M. Viquez-Zamora, H. J. van Eck, R. C. B. Hutten, Y. Su, R. Rothengatter, R. G. F. Visser, W. H. Lindhout, A. W. van Heusden

**Affiliations:** 1 Plant Breeding, Wageningen University and Research, P.O. Box 386, Droevendaalsesteeg 1, 6708 PB Wageningen, The Netherlands; 2 Solynta, Dreijenlaan 2, 6703 HA Wageningen, The Netherlands

**Keywords:** Diploid potato, Homozygosity, Inbreeding, Self-compatibility

## Abstract

Usually, mapping studies in potato are performed with segregating populations from crosses between highly heterozygous diploid or tetraploid parents. These studies are hampered by a high level of genetic background noise due to the numerous segregating alleles, with a maximum of eight per locus. In the present study, we aimed to increase the mapping efficiency by using progenies from diploid inbred populations in which at most two alleles segregate. Selfed progenies were generated from a cross between *S. tuberosum* (D2; a highly heterozygous diploid) and *S. chacoense* (DS; a homozygous diploid clone) containing the self-incompatibility overcoming S locus inhibitor (*Sli*-gene). The *Sli*-gene enables self-pollination and the generation of selfed progenies. One F2 population was used to map several quality traits, such as tuber shape, flesh and skin color. Quantitative trait loci were identified for almost all traits under investigation. The identified loci partially coincided with known mapped loci and partially identified new loci. Nine F3 populations were used to validate the QTLs and monitor the overall increase in the homozygosity level.

## Introduction

Knowledge about important characters and the genes or chromosome regions underlying the genetic variation is important in molecular breeding. In the tetraploid potato, it is difficult to efficiently exploit the genetic variation of useful traits and to different characters in a single successful variety due to the high heterozygosity at each locus (Uitdewilligen et al. 2013; Visser et al. 2014). The introduction of a new trait from a wild species may take 15–50 years, and the development of new potato cultivars usually takes over 10 years, making overall crop improvement a slow and laborious process. The lack of yield gain in potato breeding in the last century is a consequence of these technical difficulties (Douches et al. 1996). Techniques such as Molecular Assisted Selection (MAS) and Estimated Breeding Values (EBV) can possibly lead to a more rapid identification and subsequent breeding of potatoes with a superior germplasm (Slater et al. 2014).

Mapping studies in potato have been performed in both tetraploid and diploid populations (e.g., van Eck et al. 1994a, b; Bradshaw et al. 2008; D’hoop et al. 2014; Prashar et al. 2014; Endelman and Jansky 2016). In these studies, Quantitative Trait Loci (QTL) for major traits were identified. A QTL study aiming to map maturity in *Solanum tuberosum* showed a cycling DOF factor underlying the expression of the late maturing phenotype (Kloosterman et al. 2013). However, not all additional genetic variation in maturity could be explained, and other factors have yet to be identified. This was expected in tetraploid populations, where small allele dosages at other loci often complicate the mapping of minor factors (genetic noise). The traits of importance in potato include yield, tuber specific gravity (under water weight, UWW), starch content, disease resistance, tuber shape, and flesh and skin color. Tuber shape can vary from compressed to long; long tubers are suitable for French fries and round tubers are preferred for crisps. Tuber shape has a simple genetic basis, round was considered to be dominant over long, and is hardly affected by environmental factors (van Eck et al. 1994b) but recently this seems to be less clear (Prashar et al. 2014). In these studies, the *Ro* locus on chromosome 10 (van Eck et al. 1994b) plays a crucial role. In an F1 mapping population between *S. tuberosum* and *Solanum vernei*, three new tuber shape QTLs were identified (Sørensen et al. 2006), which indicated that a more distant germplasm can add novel diversity. An unwanted phenotype of tuber shape is bending, which results in curve-shaped tubers. This trait is not present in cultivated European germplasm but can be frequently found in both cultivated and wild Latin American accessions. The flesh color of tubers of potato cultivars varies from white via cream and yellow to deep yellow to red and purple. The yellow color is caused by carotenoids and red or blue/purple flesh by anthocyanins. The carotenoids in tuber flesh are thought to be health-promoting (Snodderly 1995). A diploid *S. phureja* accession (Inca Dawn) is known to have a high carotenoid level (Morris et al. 2004), and the orange flesh color was found to be caused by a recessive allele of zeaxanthin epoxidase (ZEP) (Wolters et al. 2010). Alleles of the Y locus (chromosome 3) are responsible for a yellow flesh color (Bonierbale et al. 1988). In addition to the Y-locus, modifying genes are known to give rise to different gradations of white and yellow.

To obtain F2 populations the diploid hybrids must be self-compatible. However, diploid potato is self-incompatible, inhibiting the development of homozygous lines and offspring populations derived from these lines. An approach to overcoming their self-incompatibility is to use an accession of the wild species *S. chacoense*. This accession harbors the *Sli*-gene that overcomes self-incompatibility and hence allows selfing (Hosaka and Hannemann 1998a, b). The *Sli*-gene has been crossed into diploid *S. tuberosum*, thus allowing the production of selfed progenies (Lindhout et al. 2011). This made it possible to develop F2 and F3 populations for mapping studies. After selfing diploid potato, severe inbreeding depression might occur. This may be due to the presence of recessive lethal alleles, which may hamper genetic studies because offspring plants may suffer from lethal alleles and the survivors may show a strong skewness (Lindhout et al. 2011). The *S. chacoense* parent, which harbors the *Sli*-gene, is almost completely homozygous and consequently cannot harbor many lethal alleles. Therefore, the chance to generate viable offspring progenies is higher when the very homozygous *S. chacoense* is used as one of the parents.

Until recently, F2 QTL mapping studies in potato were impossible due to the self-incompatibility of diploid potato. An F2 analysis is more powerful than analyzing crosses between two self-incompatible diploid genotypes or between tetraploid potatoes because of the limited number of segregating alleles in an F2 cross. In our study, the genes in the F2 can have only two alleles. Our study aimed to investigate how much variation for several potato characteristics was present in our F2 population and to compare those with known QTLs. Furthermore, we made F3 populations of ten different F2 plants to confirm the field QTLs found in the F2 in the greenhouse (one was omitted due to a high level of outcrossing). Additionally, the reduction of heterozygosity in individual plants was followed. Ultimately, the aim was to identify the correct tools to use for developing many good growing homozygous diploid potato lines that can be used either for targeted breeding or for making F1 hybrid true potato seeds.

## Materials and methods

### Plant material

**Fig. 1:**
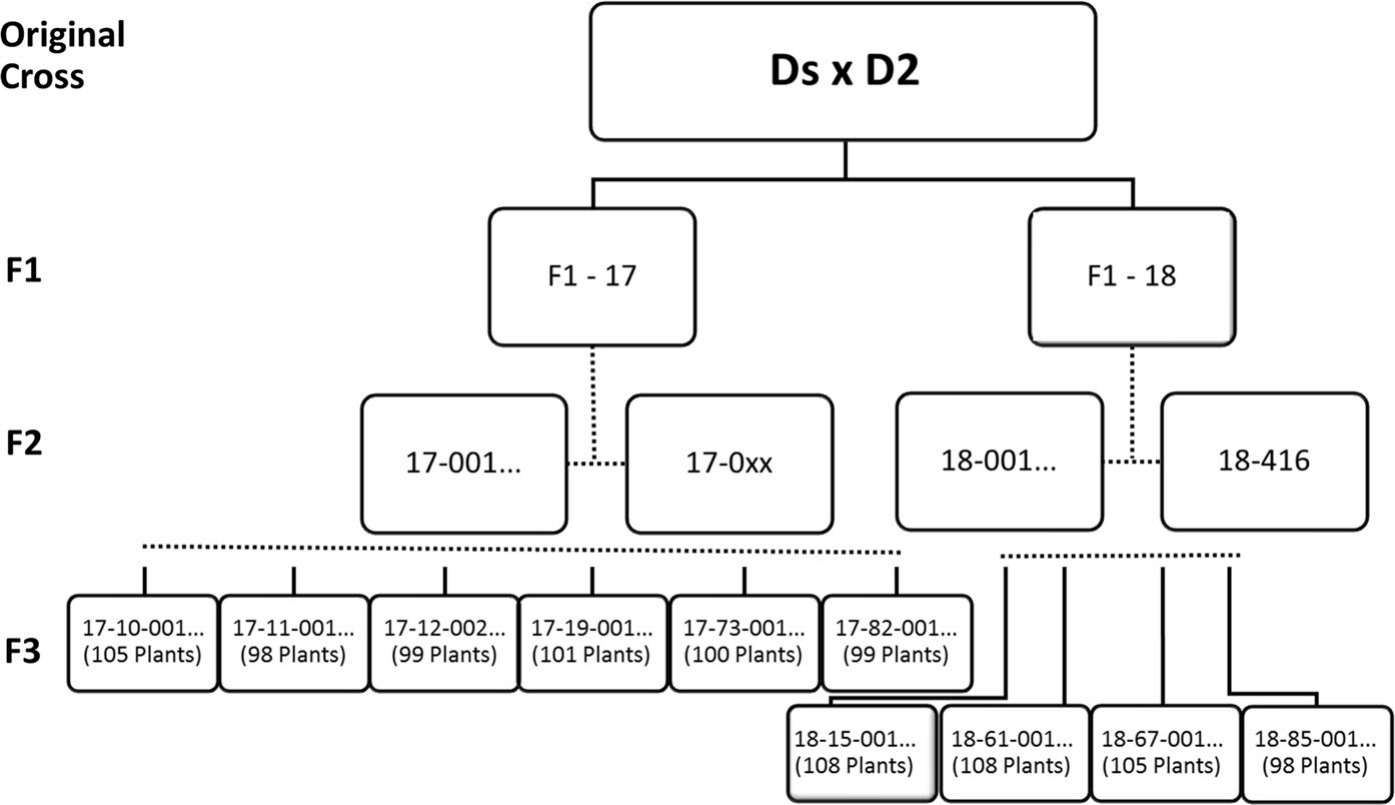
Pedigree of the F3 populations. The progeny of F1-18 was the mapping population

Two F1 plants, originating from the cross *S. chacoense* (DS) × *S. tuberosum* (D2), were chosen and self-pollinated to make F2 seeds. The two plants were part of a F1 population of many plants of which only half produced seed. The two chosen F1 plants produced seeds with a high germination rate, and their F2 populations were the most vigorous ones. A single F2 population was used for the F2 mapping study and F3 offspring of individual plants of both populations were used for the F3 studies. DS was selected because of its self-compatibility (Hosaka and Hanneman 1998a, b) and inbreeding tolerance. The tubers were long, and they exhibited deep eye depth, white tuber flesh and slight anthocyanin levels in the tuber skin. D2 was selected for cooking quality, round/oval tubers, yellow tuber flesh, shallow eye depth and no presence of anthocyanins in the tuber skin. The pedigree of D2 lacks any wild species material (Hutten 1994). Both F2 populations were grown, but only one was used for mapping purposes. The chosen diploid potato F2 population DS × D2 consisted originally of 415 genotypes; these genotypes were multiplied and six tubers per genotype were used in the field trial. The trial plants were planted on April 25, 2011, near Wageningen on clay soil in two replicates with three hill plots per genotype. Of each replicate, one plant was selected for DNA extraction and marker scores were later compared. Phenotypic scores were the average of the scores of the two replicates. The other F2 population was not genotyped but was grown and if possible, seed was collected. A total of 10 F3 populations of approximately 100 plants were selected based on the availability of seeds and growth of the parental F2 plants. The 10 F3 populations (Fig. 1) were sown on February 20, 2013, and emerged seedlings were transplanted after 4 weeks and grown under greenhouse conditions using 3 L pots in Wageningen. Temperature was maintained at 24 °C, plants were watered daily. No measures were taken to decrease light intensity during the summer months. In total, the experiment lasted 6 months, after which tubers were harvested from each plant, labelled and stored in net bags at 4 °C.

### Phenotypic evaluation

#### F2 population

Tubers were harvested after 20 weeks (September 13, 2011) and stored. Tuber phenotyping was based on digital images of the tubers. Tuber shape was recorded as the length to width ratio (L/W ratio), where the length of a tuber is defined as the distance between the apex (rose) and the place of stolon attachment (heel). The width of the tuber is the length of the transversal axis perpendicular to the longitudinal axis. The curved tuber shape was defined as a curve in the length axis of the tuber (heel to apex), and an absence/presence classification of curving was recorded in long tubers only, with an L/W ratio exceeding 1.5. Flesh color was scored using an ordinal 1–5 scale, representing deep yellow, yellow, yellowish white, creamy white and white flesh. Tuber skin color was scored in three classes: without red anthocyanin, some blush of pigmentation and with red anthocyanin.

#### F3 populations

Tubers were harvested per genotype and stored in a net bag. Tubers were weighed during the winter of 2013/2014. The evaluation was similar to that described for the F2 population. Cooking and baking quality were evaluated using a standard scale from 1 to 9, which ranged from bad (1) to good quality (9). The cooking quality was scored after cooking the potatoes without peel for 30 min and 1 day later the level of gray coloration was scored. Enzymatic discoloration was evaluated by grinding the potatoes and placing them at room temperature. Subsequently, the discoloration was scored after 24 h and evaluated for color and magnitude on a scale from 1 to 3 (1 = the lowest level of discoloration). The formation of small tubers or sprouts was scored before cooking and baking. Sprout absence/presence was scored as 0/1. The same was done for the small tubers. Stolon development was scored just before harvesting using an ordinal scale (no stolons = 0; intermediate stolon length = 1; long stolon = 2).

### DNA extraction

Leaves were collected in 96-deep well plates and stored at − 80 °C before freeze drying. DNA was extracted using the automatic KingFisher method (LGC genomics, United Kingdom).

### Genotyping

KASP genotyping was performed on the F2 mapping population with 91 informative SNP markers. These KASP assays were redesigned from other platforms such as the Golden Gate assay (Anithakumari et al. 2010), the SureSelect library (Uitdewilligen et al. 2013) and the 20 k Infinium bead array (Vos et al. 2015). All 91 SNP markers were selected because they were polymorphic between DS and D2 and because two different alleles were present in the F1. The markers were known to cover the potato genome. SNP analysis was outsourced (to Dr. Van Haeringen Laboratory, Wageningen; VHL, www.vhlgenetics.com). For the F3 mapping populations, 140 markers were chosen, of which 53–80 segregated per population.

### SNP markers and linkage analysis

The segregation ratios of the SNP markers were used to construct an F2 linkage map. Linkage analysis was performed using JoinMap^®^ 4.1 (van Ooijen 2006). The markers were named according to their chromosome locations on the potato sequence version PGSC v4.03 pseudomolecules. F3 populations were treated as cross pollinating (CP) populations with unknown phase, so the linkage map was calculated as though it was a cross pollinator with segregation type <abxab>. In a CP population JoinMap^®^ 4.1 determines the (relative) phase of the markers. The phase of one marker is set {00}, and the phase of the other marker is determined relative to the phase of the first marker. For markers with the same phase, the phase is set equal to {00}, whereas for markers with an opposite phase, the phase is set equal to {11}. An integrated map was calculated based on the combined function in JoinMap^®^ 4.1.

### QTL mapping

The quantitative data of the F2 population after harvest were tested for normality. QTL mapping was done with the software package MapQTL6 (van Ooijen 2009) using both interval mapping as well as Kruskal–Wallis analysis. For the F3 populations, a Kruskal–Wallis analysis, in combination with a physical map, gave non-parametric estimations of significant associations. Single Interval Mapping (SIM) was done based on an integrated map.

## Results

### Genetic map construction

#### F2 population

**Fig. 2:**
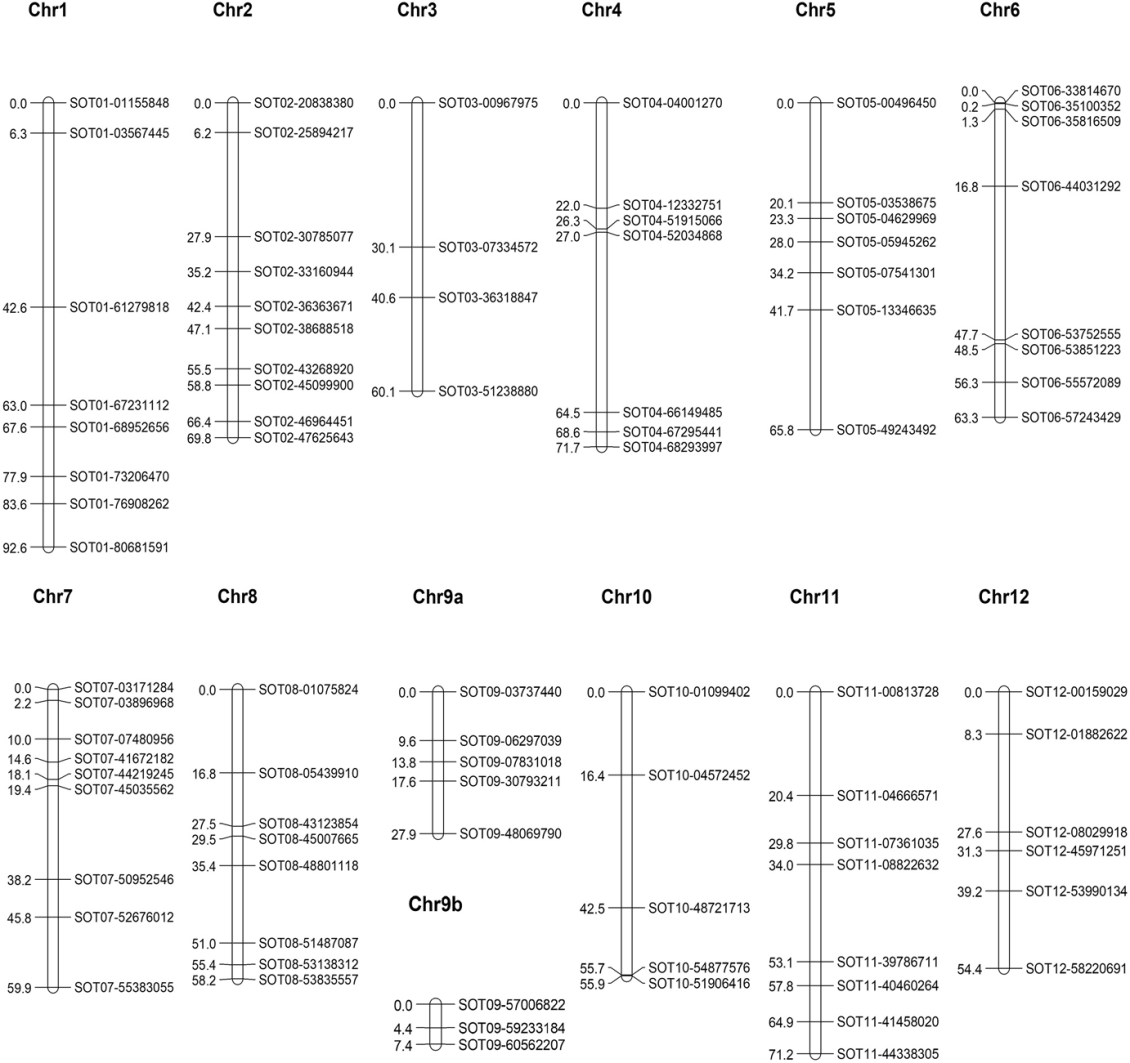
Genetic map of the F2 population based on 272 individuals and 90 SNP markers. The two genetically unlinked groups of chromosome 9 are depicted separately

**Fig. 3:**
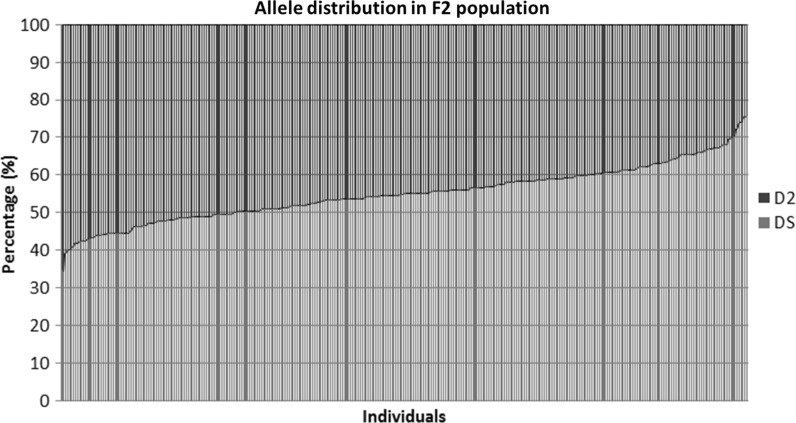
The percentage of D2 and DS alleles in the individuals of the F2 population

A subset of the original F2 population of 415 individuals was selected based on the availability of at least six tubers per genotype. This resulted in a population size of 272 genotypes. A collection of 92 single nucleotide markers (SNPs) was used to calculate a genetic map (Fig. 2). All but one of the SNPs were in the expected positions based on the physical map of DM1-3. SOT10-00139451 was removed from further analysis as it unexpectedly segregated identically to SOT08-48801168. The known locations of the markers made it easy to assign the linkage groups to chromosomes. Chromosome 2 was only partially covered because no markers were available on the short arm of chromosome 2 due to an abundance of rDNA in that region. In the genetic map of chromosome 9, a gap of 50 cM with no markers prevented the joining of the two chromosome 9 groups. The physical distance between SOT09-48069790 and SOT09-57006822 is only 7.6 Mb, so this region must be a hotspot of recombination. On chromosome 10, the SNP markers SOT10-51906416 and SOT10-54877576 mapped so close together that their exact order could not be reliably calculated. The percentage of DS alleles in an individual F2 plant ranged from 35 to 75%, with 192 genotypes (71%) possessing a surplus of DS alleles (Fig. 3). The distribution of loci in the F2 population homozygous DS vs heterozygous vs homozygous D2 was 7537:15,870:4578, with 45% D2 alleles and 55% DS alleles. In particular, the segregation of markers on the short arm of chromosome 1 and the long arm of chromosome 12 was heavily skewed in the direction of DS. Only for chromosome 9 was there a preference for D2 alleles (Table 1).

**Table 1:** Allele distribution per chromosome. Number of alleles homozygous DS, heterozygous H and homozygous D2 were calculated per chromosome

Chr.	DS	H	D2
1	631	1524	271
2	1158	2530	620
3	1513	3239	903
4	608	958	318
5	575	1107	200
6	547	1156	455
7	802	1615	542
8	498	1194	464
9	403	1406	601
10	311	778	251
11	761	1571	358
12	888	1322	205

#### F3 populations

Self-pollination of the F2 plants was stimulated by hand pollination and was repeated if no berries were formed. Flowers were not individually bagged in the field. Therefore, cross pollination is a possibility. Comparing the marker patterns in an F2 plant with the marker patterns of its F3 population clearly shows which F3 plants are the result of cross pollination (a considerable number of homozygous markers in the F2 plant will be heterozygous in an F3 plant in case of a cross pollination). We removed these few plants from all populations. Only in one F3 population (18-061) the level of cross pollination was too high and therefore the number of remaining self-pollinated plants too low to perform mapping studies. Skewness in the direction of DS was found on chromosomes 11 and 12. In the F2–F3 inbreeding step, no further preference for DS alleles was found (the remaining heterozygous loci from the individual F2 plants segregated in a normal Mendelian 1:2:1 ratio). The homozygosity level of the three remaining F1-18 F3 populations was 70%. The average value homozygosity of the six F3 populations originating from F1-17 was 76%, but a preference was also found for DS alleles in these populations (on average, 62.5% DS). However, without genotyping data of the F2 parental plants originating from F1-17, it is not certain that this preference for DS alleles was present in the complete F2 population.

**Fig. 4:**
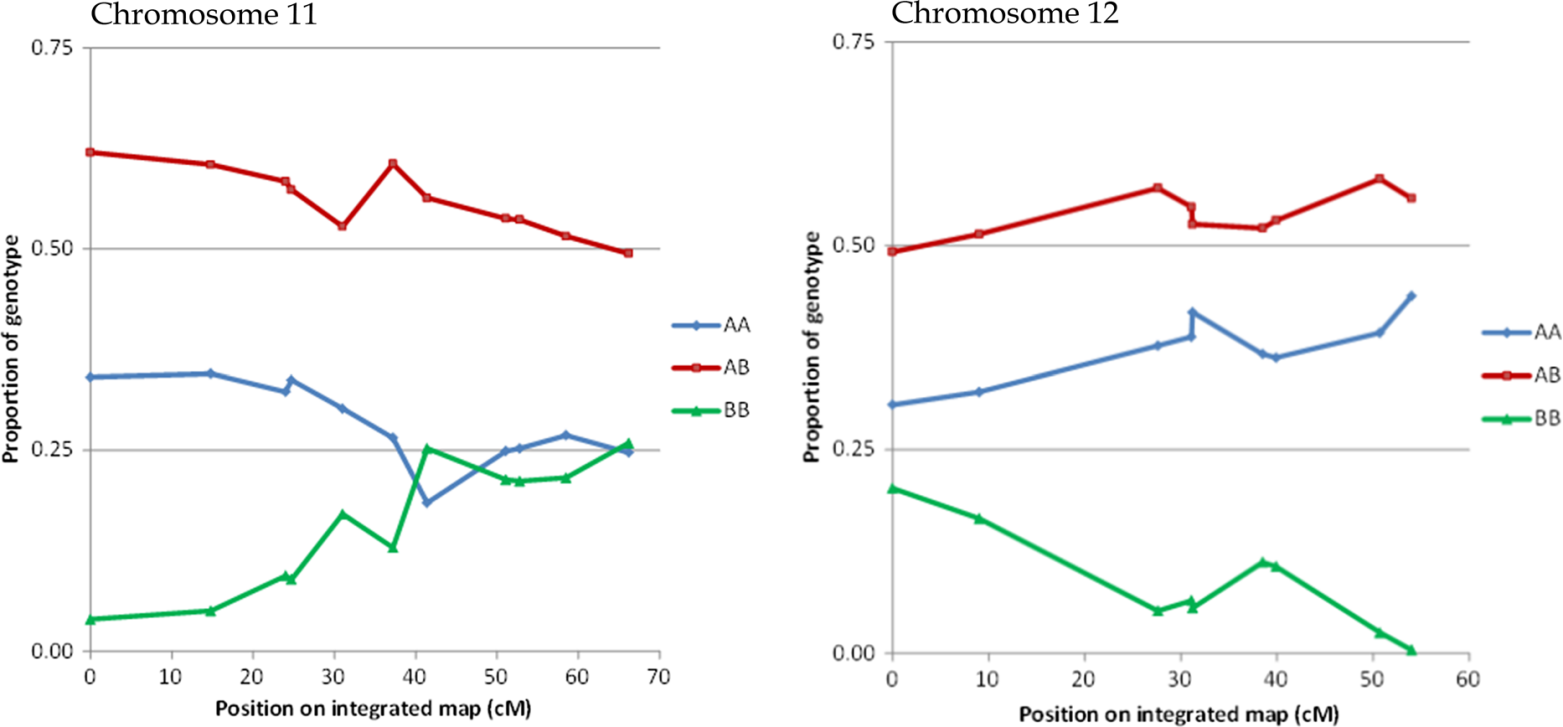
Skewness on the top of chromosome 11 and the bottom of chromosome 12. AA = homozygous DS, AB = heterozygous and BB = homozygous D2

For the nine remaining F3 populations, individual linkage maps were calculated by using JoinMap^®^ 4.1, and an overall integrated map was calculated. Two chromosomal regions (on chromosomes 11 and 12) showed a skewed segregation pattern. For the top of chromosome 11 and the bottom of chromosome 12, almost no homozygous D2 genotypes were found (Fig. 4).

### QTL mapping

#### Tuber shape

**Fig. 5:**
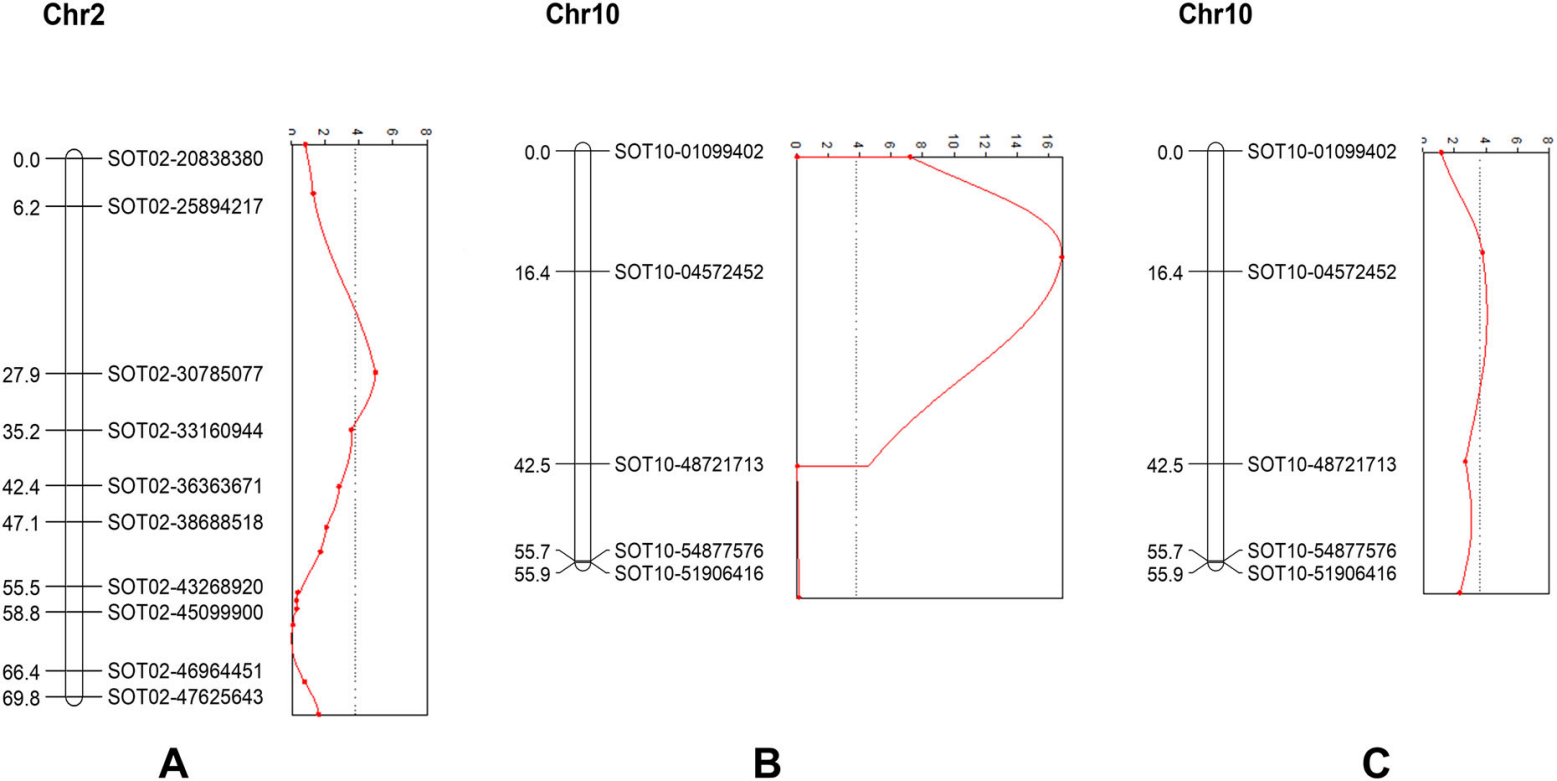
Interval mapping: tuber shape (**a**, **b**) and curved tuber shape (**c**)

In the F2 population, the length/width ratio of tuber shape varied between 1 and 2, with an average of 1.56. A major QTL for tuber shape was present on chromosome 10, and a minor QTL was on chromosome 2 (Fig. 5a, b). The major QTL had the peak marker SOT10-48721713 (LOD 16.9), which explained 26.1% of the total variance, with the round tuber shape dominant over the long tuber shape. F2 genotypes that were homozygous or heterozygous for the DS had an average L/W ratio value of 1.4, and F2 plants homozygous for the D2 allele had an average greater than 1.9. The minor QTL near SOT02-30785077 (LOD 5) on chromosome 2 explained 6.3% of the variance, and the longer tuber shape was dominant over round. For chromosome 2, the alleles conferring long tubers originated from DS. The QTL on chromosomes 2 and 10 were also clearly present in the F3 populations. Unexpectedly, the position of the QTL on chromosome 10 in the F2 shifted somewhat to the end of the chromosome in the F3.

#### Curved tuber shape

For curved tuber shape, a QTL (LOD 3.8) was also found on chromosome 10, near the marker SOT10-48721713 (LOD threshold 3.5) and explained 18% of the variance (Fig. 5c). The allele enhancing curving originated from DS. No measurements for the curved tuber trait were done in the F3 populations.

#### Tuber skin and flesh color

By using a Kruskal–Wallis analysis on the F2 data, several QTLs for skin color were identified (Table 2). The QTL with the strongest effect was located on chromosome 2, with a peak marker SOT02-39604838. Additionally, minor QTL were found on several other chromosomes. A major QTL for flesh color was found on chromosome 3 near SOT03-00967975. In the F3 populations, the flesh color QTL was clearly confirmed, but the position of the QTL shifted more to the center of the chromosome.

**Table 2:** Location of QTL regions identified

Trait (interval mapping)	Peak marker(s)	LOD	LOD threshold	Expl. variation	muA	muH	muB	Literature
Tuber shape	SOT10-04572452	16.9	3.8	26.1	1.38	1.45	2.09	Prashar et al. (2014) van Eck et al. (1994a, b)
	SOT02-30785077	5	3.8	6.3	1.94	1.67	1.6	
Curved tuber shape	SOT10-04572452	3.8	3.5	18	0.21	0.18	0.95	

Trait (Kruskal–Wallis)		Significance	Nr inf.				
Flesh color	SOT03-00967975	*******	241	4.05	2.86	2.7	Bonierbale et al. (1988) Wolters et al. (2010)
Tuber skin color	SOT02-39604838	*******	263	0.12	0.49	0.97	van Eck et al. (1994a, b)
	SOT05-13346635	******	261	0.65	0.40	0.11	
	SOT06-35100352	*****	262	0.72	0.30	0.39	
	SOT09-07831018	******	253	0.51	0.56	0.1	
	SOT10-04572452	****	264	0.56	0.48	0.02	
	SOT11-41458020	**	265	0.63	0.42	0.25	

The asterisks stand for the level of significance **0.01 level; ***0.001 level; ****0.0001 level; *****0.00001 level; ******0.000001 level; *******0.0000001 level. Nr inf are the number of plants used for the analysis

## Discussion

### Linkage mapping in self-compatible potato

SNP marker names were based on the chromosome’s number and position on the PGSC *S. tuberosum* group Phureja DM1-3 Pseudomolecules (v4.03). The new names made it very easy to show, on the F2 genetic linkage map, that the 91 markers were in the expected positions based on the physical map. This result shows that there is no disagreement with the potato physical map. A comparison of genetic and physical distances showed, as expected, a very low level of recombination in the region surrounding the centromere (Fig. 2). Since one of our parents (D2) was partly heterozygous, we screened the markers for DS, D2 and the two best F1s and selected only markers with two alleles in the F1. The integrated linkage map of the F3 populations corresponded to the positions of the F2 map (data not shown). The positions with the strongest skewness in the F2 population coincided with the genetic positions of the S-locus on chromosome 1 and with the HT-B locus on chromosome 12. These loci are known to play important roles in the gametophytic self-incompatibility mechanism (Marcellán and Acevedo 2014; Tovar-Mendez et al. 2014). In the combined F3 populations, the segregation ratio on chromosomes 11 and 12 was very skewed in the direction of DS. For chromosome 11, this might have been due to the F2 plants that were used as parents for the F3 populations that were not genotyped and could, by chance, have been homozygous DS for these chromosome regions. In the F2 generation, only 7 plants were found with homozygous D2 alleles on the bottom of chromosome 12 (only 7 plants homozygous for D2 for marker SOT12-59211472). It is tempting to connect the skewness on chromosome 12 to the position of *Sli* on the bottom of chromosome 12 (Hosaka and Hanneman 1998a, b), but that would imply that *Sli* not only induces self-compatibility but is also needed for growth because all F2 and F3 plants were genotyped (with or without seed set). In the three F3 populations of F1-18, no D2 homozygosity was found on the bottom of chromosome 12, although the parents were heterozygous in this region. Overall, there was a clear bias in the F2 population towards DS alleles; approximately 55% of the alleles originated from DS, but this was not the case on chromosome 9. This bias towards DS did not persist towards the F3 populations, where the overall level of homozygosity increased to the expected values based on the parental F2 value (Table 3). In the F3 populations, individuals were already present, with over 90% homozygosity based on the 140 markers that were heterozygous in the F1. The overall higher percentage of DS alleles might be due to the self-compatibility of DS. DS has been subjected to multiple rounds of self-pollinations, which might have led to more alleles with a selective advantage. The number of deleterious alleles reduces with each cycle of self-pollination because fixation can be lethal. D2 had not been subjected to self-pollinations and might consequently contain a larger number of deleterious alleles.

**Table 3:** % DS is the percentage of DS alleles (homozygous DS is two alleles). Homozygosity average is calculated for homozygous DS and D2 genes

Population	Average value % DS in F2 parental plant	Range and average value % DS in F3 populations	Homozygosity range and average in F3 populations
SOL013-3010 (F1-17)		47.1–67.9 (58.5)	64–92 (75)
SOL013-3011 (F1-17)		45.6–67.5 (58.6)	65–89 (76)
SOL010-3012 (F1-17)		45.6–70.8 (60.8)	54–89 (75)
SOL010-3019 (F1-17)		54.4–71.5 (63.4)	68–92 (80)
SOL010-3073 (F1-17)		62.0–79.2 (70.3)	60–85 (72)
SOL010-3082 (F1-17)		52.9–75.2 (63.1)	64–90 (78)
SOL010-4015 (F1-18)	60.6	47.1–70.1 (61.4)	57–86 (72)
SOL010-4067 (F1-18)^a^	64.2	54.4–75.2 (64.8)	53–86 (71)
SOL010-4085 (F1-18)	52.6	41.6–67.5 (54.3)	51–82 (67)

^a^SOL010-4067 was discarded due to an excessive level of cross pollination

### QTL mapping in self-compatible potato

#### Tuber shape

The two QTL detected for tuber shape on chromosomes 2 and 10 have been identified before (Van Eck et al. 1994b; Prashar et al. 2014; Endelman and Jansky 2016). Van Eck et al. (1994b) observed a heritability of 80%. Thus, we expected a higher explained variance than the 24.2% that was observed in the present study. This lower explained variance might partly be caused by the contribution of the minor effect QTL, but the relatively low marker density in this region could also be why the locus with the highest explained variance was not precisely identified. The tuber shape QTL on chromosome 10 was also associated with eye depth (Prashar et al. 2014), and in our studies, it was shown that a QTL involved in curved tuber shapes was likely a QTL on chromosome 10. The co-association of a curved form and tuber shape QTL at the same SNP locus is remarkable and could be an artifact. Tuber shape is epistatic over curved tuber shape, so the curving might be caused by tuber length variations within our material. To discriminate between the hypotheses of closely linked genes and genes with pleiotropic effects, a self-compatible diploid should be backcrossed, and recombination screening should be performed to identify the most likely hypothesis and, eventually, the candidate genes for this important potato trait.

#### Tuber skin and flesh color

Several QTL for skin color were identified by using the F2 population (on chromosomes 2, 5, 6, 9, 10 and 11). The QTL with the strongest effect was on chromosome 2, with the peak marker SOT02-39604838. Flower color genes were mapped to chromosomes 2, 10 and 11. These positions are consistent with the literature and most likely represent locus R on chromosome 2 (van Eck et al. 1994a). The QTL on chromosomes 10 and 11 most likely represent the I and P locus (van Eck et al. 1994a). A major QTL for flesh color was found on chromosome 3. This finding is a validation of studies revealing *Chy*2 or *Bch* on chromosome 3 as the causal agent of yellow flesh in potato tubers (Brown et al. 2006). No new QTL influencing flesh color was identified in our study. The QTLs for tuber skin color in the F2 were not confirmed in the multi-population analysis; however, a Kruskal–Wallis analysis on individual F3 populations confirmed four of the six QTLs found in the F2. In the F3 populations, the flesh color QTL was clearly confirmed on chromosome 3, but the position of the QTL shifted more to the center of the chromosome; this was also seen for the tuber shape QTL on chromosome 10. This might have been due to the calculation of an integrated map, where the random choice of individual F2 plants might influence QTL calculations on the integrated map in F3 progenies. Therefore, we think that the positions on the F2 map are more trustworthy.

#### Cooking type

For cooking type, a candidate gene with homology to a tyrosine-lysine rich protein (TLRP) was identified based on allele specificity of the probe on a microarray (D’hoop et al. 2014). TLRP was mapped on chromosome 9 at a position associated with this QTL for potato cooking type.

Only one previous mapping study in self-compatible diploid potato has been published (Endelman and Jansky 2016). In that study, they crossed an S7 of *S. chacoense* (containing *Sli*) with the doubled monoploid DM1-3. Ten associations were found, seven of which were already known. In our study, we confirmed the possibilities of F2 mapping in potato and the development of further viable offspring populations. To create mapping populations with a maximum of two alleles per gene, the diploid potato must be self-compatible, and inbreeding should not result in too many unexpected segregation ratios in the mapping populations. The transition from self-incompatible to self-compatible in diploid potato can be done by the introgression of the S-locus inhibitor (*Sli*) gene (Hosaka and Hanneman 1998a, b). By repeated selfings of self-compatible diploid potatoes, more homozygous lines can be obtained (Birhman and Hosaka 2000; Phumichai et al. 2005). These authors showed that the level of heterozygosity was decreasing and that no chromosomal regions could be identified that were exclusively heterozygous. A high level of inbreeding depression was associated with the selfings in the study of Birhman and Hosaka (2000), but this was less the case in the study of Phumichai, who showed that the background of the diploids might play an important role. The success of making self-compatible diploids depends not only on *Sli* (as the pollen donor) but also on the mother plant (Phumichai and Hosaka 2006) and might be related to differences in the chloroplasts. The Hosaka group developed progeny populations up to S9, but heterozygous regions can still be found (http://www.obihiro.ac.jp/~Potato/research/Inbreeding.html).

Overall, we have shown that the F2 and F3 mapping populations can be used for mapping purposes and that our results confirm those obtained in other populations. We show that progeny populations can be developed for more detailed studies on specific traits. In the F3 populations, only one region was found in which no D2 alleles were found. This result might be partly coincidental because in the F2 population, it was very skewed but not absent.

### Future perspective for potato breeding

Potato is considered a tetraploid crop. However, with the introduction of self-incompatibility blocking mechanisms, such as *Sli* from *S. chacoense*, a diploid potato crop has become increasingly attractive because of the more efficient breeding, which results in a constant improvement of cultivars. This is achievable because the self-incompatibility mechanism has been broken/overcome, so the fixation of alleles is thus a real possibility. An efficient development of diploid F1-hybrid cultivars also requires a profound knowledge about the genetic and phenotypic factors involved in vegetative and generative development. Self-compatibility in diploid potato will lead to more homozygous lines with fewer deleterious allele combinations. Such diploid homozygous potato lines can be used for developing F1 hybrids, making specific crosses, the extensive mapping of important genes and studying heterosis and combinability. Extensive mapping using recombination populations will allow a better location of major genes and will facilitate gene cloning. With non-segregating major genes, it will also be possible to focus on minor effects. The underlying factors influencing complex traits in hybrids, such as tuber yield, can be studied in more detail by developing targeted mapping populations such as recombinant inbred lines. To obtain more homozygous, self-compatible diploid lines, Solynta (www.solynta.com) is setting up a mature breeding program with a selection of the best individuals in 750–1000 crossing diploid combinations every year and picking the best combinations to use as parents for selfings or crosses with other good parental lines. The first hybrid field trials have shown that diploid F1-hybrid breeding is making significant progress (De Vries et al. 2016).

## Appendix 1

Marker name including chromosome number and position (position according to their chromosome locations on the potato sequence version PGSC v4.03 pseudomolecules) and flanking sequence of SNP (SNP is between brackets).

SOT01-01155848 CATACGAATGAGGGCCAAATCTGCTGGACTGCCAACAGTTGCACCAATTGCTCCAGCAGT[T/C]AGACCACACAGAGCCTTTTGGTACAGAGGTAAGGGCTTCCCTTCATTAGCCTCAATGGCC

SOT01-03567445 CGGAATGAATCAGAAAACATGGCCGCCGACCAAGTCCAATTCCGACGGCC[C/T]TCGTCGTTTCACCGCTTTCCCTTTCATCAGAAAACAGACCCATCTTCAAA

SOT01-61279818 TTTATCAGCTAGTATTAATTCTTATTTATACTGTGTGATTGGTTTTTGCAGGATATGAAG[C/T]TCCACTTTTGAGGTAAGTACAGAGTTCCCTGTCTCAGATTTGATTCAACCCTATTTATTA

SOT01-67231112 TGGTACCTTTCTGAATCTGTTTCCTTTCACTTTGTAGCTTGAAGTCAGGGAAACTACGAT[T/C]AACACGCAAGCGTAAGCGGAAGTTTGGGGCTACACAGACTCAACCCAAGAAAAAGCAGCA

SOT01-68952656 AGACACCTTAAAAGATAAGCGGATATATGGGTCTCTTTTAAATGCCTTTGTGCGGTCTAG[A/G]AAGAAGGAACAAGCTGAATCTTTACTCGATAAAATGAGAAACAGAGGCTACACAGATCAT

SOT01-73206470 ATCGGGGTACAACCCGGAATCTGAATCGGGTGGGGCAACTCTCTGTACTCACACGACACC[A/G]TTTCATCAAGCTTCGGCAAGTAGAGAAACAGAGACAAAGCCATAGCAGTAGAAGGGAAAA

SOT01-76908262 TGCACGATCTCCTCTTAAGCTTGTTACTAGATTTCCCTATCATCCTGAAATTGGGACACT[G/A]CACGATAACTTTGAGTAAGAATCTCGTCTTTAGCTTTTTACTTTTAATTGCTTTAAATAT

SOT01-80681591 TGTCATTATCTCTTATCACTTCAATTGAGTCAATTTCTGATCCTTCTTGATCCGTAACCG[A/C]TGCATTTGTTGCATCAAATCCAAATTTCTGACCTGTACAACACGTAAATACATTAACACA

SOT02-20838380 GGGAGGAAATCCAGCTAGGTTTCTGAGGAAGCTCACACAAGAAGAAATTGCTTTTATCTC[A/G]GAGTCAGCAGCCAACTATTCTAATTTAGCACAGGTCCATGCTGGTGAAAATGCAAAAAGC

SOT02-25894217 ATGATACACATACATGACATGAGTGGCAACTTTCTAGCAATTCAGCACACCTAAAATTCA[T/C]ATTTCATCTACACATGGCGGGAAGTAATGAGCTAGTTTCCGGTAAGGTAAAGTCACCTTT

SOT02-30785077 GTACATGCCATTTTATCTATCCCTTGCGACTTTTCTCATGAGCCTTTCTTTCTTTGCCTA[T/C]GGAATGTTCAAGAATGATCCATTCATCTCTGTAAGTAAATATTCCATTCTTCAGGCTGAT

SOT02-33160944 AGCAGGCCACCTGTTTCCATGGTTTGGAACCTGAATACCGACAAAAATACCAGTCCTACC[C/T]TTCTCCTGCATTTGGATACCAGCTTGAGCTCTCCCATAAGGAGGGCTTGTAAAAATTTCT

SOT02-36363671 CATAGATCATTGAGTTGCACAAGTGTCTCCAATGCAATAACCCACCTCAAGATCAAACCT[T/A]TTCTTGGGTTTGTCTCTCATGGAACCACAAGTCTATCAGTACAAGTAAATTTGTCTTCTA

SOT02-38688518 GCTTCCATTGTGCATTGTTTTACTCTGCCAGGGTTGCCTACGCGGCATGA[A/G]GATGTGGATATTGTTGTGATTAGGGAGAACACTGAGGGAGAGTATGCTGG

SOT02-43268920 ATGGTTAACTCAAAAGACAACTCCTCTACCGACAGCGAAAACTCCTCCGGTGACGTTCCC[A/G]ACTCTAAACCTAGTCTCTACTTTGAAGGCGAGAAGGTTCTCGCTTATCATGGCCCTCGCA

SOT02-45099900 TGCTGGGGAAGTAGTCGATGACTACTTGGATCTTGCAGAATATGGAGGGGATAGTCAGTT[T/C]AATGATCAGTACAATGTTAACCAACAGCAACAACACTACTCTGTTCCTCAGAAGAGCTAT

SOT02-46964451 GAGGCAAGCATCTCGAGCTCTGAAGGAAGGGAAAACCTTGATTGTTTCAGGAGTAGCTAA[G/A]TCAAAGACTGACCACGCTATTGAATTGCTAAACAAACTAGAAGCTGGGTTAGGTGAGCTT

SOT02-47625643 CTTCAGCTGCCCTCTCCTTCGAATTCCAATGCAGAACTTCAGACGCCAAGCATCAACCAT[T/C]GATTCTGGTGATGAATATAGCAAACCCCACAGAAGAGAATGGGGCCAGTAAGGTGTGCAC

SOT03-00967975 TCATCCAGATTTCCCCACCATTTGTACATTCCTGGGAGCTCTATAAGTGC[G/A]CCTGTTACTCCACCTTTGAGCTCTCCCACAGCTCGTACACCTAGAATGAG

SOT03-07334572 GAGATTGGTGCTGAAGCAGTTGTGTTGGCACTTGGGCCTTGGAGTAAGAG[A/G]TTTTCTGTTTTGGGCTCGCTTTTCAGGGTTTATGGAATTAAAGCCCATAG

SOT03-36318847 AATGCAAAAGCAAGATCCTAGTTACAGCAGTCCTTCACTTCTTCCCTCTCTCCTTGACTA[T/C]TTTTGAAGAGCATTAGACAAAATCAGAGCTCATTAGCCACTGTGAAAGAGAGATAAAATC

SOT03-51238880 ATGCTCTGTTTTGGGTAGGTGAAATTGGGGCTAATGACTATGCGTATAGCTTTGCATCTA[G/C]TGTTTCACCCAACACAATTCAACGGCTTGCCACCAGAAGTTTCACTGGTTTTTTACAGGT

SOT04-04001270 ATTGAGTTGGAAGAGTTCCACCGGAGTGGATATACCATATTAGACTGCGTTGGCATGTGT[G/A]CTTGTCTATATACATTCGGAAGAATCAGTTTGTAGGGAGTGTAATGTTTTGAGGAGCTGA

SOT04-12332751 TTTGAAGAACAATGCTGATCTCAAGGAGAGAAATAGTAGAATACCAACTTTTCTTTATGC[A/C]ATGCCATTTTCATCCAACAGGATATTTCTTGAAGAAACCTCACTCGTAGCTCGTCCTGGT

SOT04-51915066 GAGTTTAAGGAGAACAACAAAGTGGACAAGGTGGTGGTTTTGTGGACTGC[T/C]AACACTGAAAGATACAGCAGTGTGGCTGTTGGCCTTAATGATACCATGGA

SOT04-52034868 AAACTGTGTAATGAACATAAACTAGAGAAATTAGTACCTTTTAATTTATGACAAATAGCC[A/G]ATCGAAGTTCCATTGTACCTGCATTTGGTGTATACCTGGTATGACCTTCACGAATTGCAT

SOT04-66149485 TCTGCTAACCTGCACTCTTTGCTATCTTTGTGCATTTAACCTTCATGACAGACCTTTGTA[T/C]TTGGCAACCATTTTATCATTTGTCTATGCCTTCGGTCATTTCTTGACAGAGTTCTTGATC

SOT04-67295441 CCCATCTTTTTCCATTCCTATGGAAACGGCCTGGGGTGAACATGCAGATAGTAGTACACC[C/T]ACCGTGACCAGATACACAAGGACTCTAACAAATGTGGGAAACCCAGCTACATACAAGGCC

SOT04-68293997 ATGACCAACATGGATAACTACCTCCATTGCATTCTTAATTTGAACAGGATCTGGCAAAAA[C/A]CGAAATGCAATTCCTTCAACCTTCAACATGTAAACCTGCAAATTGATACAAAAAAGAAAG

SOT05-00496450 AAATGTTACCAGATCTGTTTGAAGAGTAACTTGAATGGGTTCAAACTGAGTTATAAAGGG[C/A]AGTTTCATATGAAAACCTACACATATAAACATACATGTTTGGTCAACAATAGACTTCAAA

SOT05-03538675 CCAGAGATTCAGTTGGGCGCCCATACATTCAGAAACCAGGGAGTGGAAGTAGCAAGATTT[C/T]ACATGCATGACTGGCTTATTCTCTTCCTACTTGTGGTGATAGATATAGTTTTAAATGTGA

SOT05-04629969 TTTTGGGATTTGCTTTGTTGGTCTTCTTGCAATGGTGTCATCTGTTTATGGTTATGGTGG[T/A]GGAGGTTGGATTAATGCTCATGCAACTTTCTATGGGGGTGGGGATGCTTCTGGAACAATG

SOT05-05945262 ACGGGTTAACTGATCCTTGGATGTACTCTCAAATATCTTGTCAGCATTTCCAACTTCAAA[T/C]CCGTCCCGCCTATGAAACTCCTCCACCTAAAAGATGTATCAAAATATAGTTGATCAAAGA

SOT05-07541301 CCAGAATCTGGCGTGCAGCGGTGCGGCCAATTCCATGAATGTATTGCAAAGAGAACTCAA[C/G]CCTTTTGTTGTTTGGAACCTCCACTCCTCCCACACGCACACACTTTATAACCAAACCTCC

SOT05-13346635 GATGCTCCAAGCATGGACTGACTTGGCTTTAGCCCTCACTACATATGTGGTTAAATTATA[A/G]TGTCTGCAGCTCATTTCATGGGAAACCCACTACACACCCATAGTTGATATGATGCGACTC

SOT05-49243492 ACGTTCATCAAGAGTCGAAAACTAGCATATGATGTCTATAGGTTGGATATTTATACCAAA[A/C]GTAATTGCCCAACATATTCACATACAGATCAATTCATACCTCAGAAAGTAGCCTCTCTCT

SOT06-33814670 GCTTGTTATGAGCTCACTTGCAACAATGCAGCTCAATGGTGTCTCCAAGG[A/G]ACTATTACAGTCACTGCAACTAATTTTTGTCCTCCGAACCCGTCTCTGCC

SOT06-35100352 AAACATCTATTTAGGTACATCTGTGGGATCTGCATTTATCTGATCTTGATGAACAACAAC[A/G]CATTTCAGGGATCATGCTGGAGGAAATGATAAAATAAAGCAGCTCGTTCCTGGAATAAAA

SOT06-35816509 CACCCATTGGACCGCCAGCATATGCAACAATATCACCAACTTTCCTCCCAGTCAATCCGG[G/A]ACCAACAGCTGTTACAACCCCTACAGCCTCCATACCTGGCATAAAAAATGTAATTCTAGC

SOT06-44031292 TTTGGTACGATGAGGGAAGTCCACATAGAGCGTACGACCCGAGTTTCTTGTACTCATCTG[A/G]ATTTGAAGGTACAAGGGTTAGGTATTCTGCTGGGATGATTTGGTCAGTGTCGATATTATC

SOT06-53752555 GCTCGCCGGGATTCTGAAAACGAACGGCGTACAAGCCGACGGAATCAATTTTGACAAGGG[T/C]GCCAGGACGGCTTTGGCGTTCGTGACTCTACGCGCCGACGGAGAGCGTGAGTTTATGTTT

SOT06-53851223 ATTAATGTCAATTCAGGACCGACAATCAGGTGATGATGAAGCTATGGCACTGGATGAGAA[T/C]TTTTGCACAGCTCTTGAATATGGATTACCTCCTACTGGTGGTTGGGGATTGGGTATTGAC

SOT06-55572089 AATGATGCTGCTGGTGACGGGACAACAACTGCATCCGTTCTTGCCCGGGAAATCATTAAG[T/C]ATGGTCTTTTAAGTGTTACATCTGGTGCAAATCCAGTCTCTCTGAAGAGGGGCATTGACA

SOT06-57243429 CTTGTCCCTGATTTCTCCCTTTTGCTCGAGTTGTCGGCCTCGGATGATATTAGAAACTTC[C/A]AAAAGGCTGTAGAAGAGGAAGGCCATGACATCAATGAGGTGGGCTTATGGTATGCTAGGA

SOT07-03171284 TTGATCCACCGGCATTATGCAAATTACCAAAAGTACTCACATTACTCTGCCCCCCAACAG[C/T]ATGATTCTTAGCATTCCTCCCTTCATTCGCCCCAGAGGTTGGACAAGTAAACATGGGCGA

SOT07-03896968 AGTATCAAAACATACTACGAGTGGACAAAACATTACCTGAGTGCTTGGGACCGAGATGCC[A/T]GTACAGGGATCACATTAATCCTACTCCCATTTTGACAATAAACAGCGTGACAACGAAGTG

SOT07-07480956 CTTTCGACCAAGAATTTGATGTTACATGGGGTTATGGGAGGGTGAAAATA[G/C]TCGAAAACGGGCAACTTCTCACTCTTTCCCTTGATAGAAGTTCTGGCTCT

SOT07-41672182 TCCTACGGATAAATTGGCTGTGTATCTGGCTGATGATGGAGGATGTCCTTTGTCATTGTA[C/T]GCCATGGAAGAAGCATGTGTGTTTGCAAAGCTGTGGCTACCTTTCTGTAGGAAGTATGGA

SOT07-44219245 TTCGAAGTACCCAAATTTCTACTGCTTCAGATGGGAATAAGGAGAGTGTTTCTGAGCACA[T/A]TGATGCTGGAGAAGATTCTCTTCCAGATTTAAGTCAAATTTCCAAGGATGCTGACAGCAT

SOT07-45035562 TGCCAAGAAACCACCTAGAAGACCAAGAAGATTAAGTGTCCCCATGAAATTGGTGACAAT[G/A]TTAGCAGAATTGGAGGATGGAAGATGTAAATCTCCAACCAAGTATGTGACTAAATTCATC

SOT07-50952546 TATGAGTTTGCCATCATGTTCAATCTCATAACCTTCATCCTTCACTTCATGAGATCGGAC[C/A]ACTAAATCTGCAAGCATCCACACATGTTAACATCAAATATATGTGCTAAAATCCATCAAG

SOT07-52676012 TCTGTATTGGCCAAAGACATTCTTCTGCTGCCATTGATTTGGAATAAAGAATGACCGATA[C/A]AATGGAGGAGATAGTTCCCTGTCCAGATATGGAAGTGCTGCAACAATCTGTAGGCAAAAC

SOT07-55383055 AGAAACAGATATAATTAAATCTGTTAAAGCTGATTTAAGAGCTGTTAAAGAAAGGGACCC[T/C]GCTTGTATTAGTTATGTACACTGTTTCTTGAATTTTAAAGGGTTTTTAGCTTGTCAAGCT

SOT08-01075824 TATGATTAACAAAACTATATATGTACATAATACTTAATAGTAGGAGCTTTTTCTCTACAA[T/C]ATTAAATGTGGAAAGGCAAAATTTATTATTATTTTGTACAAAGAAAAAGAATATACTCAT

SOT08-05439910 AAAAGAAAAAAAAGGTCACAGACCACAACCAAATTTCTGCCTCTTCTTCTAAGTAATCCA[C/T]ACAAAGTGAATGAGAAGAAAGAGTGTCTATATGCAAATGTAAATTGTTTTCGATGGTGAG

SOT08-43123854 GGATCAGGCTTCATACCACTTTCTTGCACTTCAGACATAATGGAATGGATATAATTAAAA[C/T]GCTTCTGGACTGTCAAAGCTGCTAGGAGGGTAGTGTATGTAATTAGAGATGGTTTATGTC

SOT08-45007665 CAGTAGGATTTATATGCTGCCACTTTGGTTAGGCTGCTCCAGTTCTTCCAGAAACTCCAT[T/C]GTCATTTTCATGAAGCTTTGCTTGTGCAATCGTTCCCATTCCTTTCAAGGCATTTTTTTC

SOT08-48801118 GTAATTGGGTTAGCAGCTGACTCTGGTTGTGGTAAAAGTACATTTATGAG[A/G]AGATTAACAAGTGTTTTTGGTGGTGCTGCTGAACCACCAAAAGGTGGAAA

SOT08-51487087 TACACCTCTAAACGGTGTCGTTCATGACTCACCTGGTGATACACCTGATCATCCTGCTGT[T/C]GGTGGTGGCTCTGCTGATGGTTATGCTTCAGAGGATTTTGTTGCTGGTTCTTCATCTAGC

SOT08-53138312 AACAGAACTTGACGAAGCCCAAACAATACTAGGTTGTGGATTAGCCATTTTACATGCTTC[A/T]AGCAAACTAACAAAACCAGCAATATTACTATGAACATAAGAACCTGGATTCTGCATTGCA

SOT08-53835557 CGAACCGGTTGGATTGGTGGCTTACTCGGTAAGCTCTGTGAAGCTCAAGGCATAGAGTAC[A/G]AATACGGGTCGGGTCGATTAGAGAATCGGAGCTCCTTAGAGTCCGACCTCACCACCGTCA

SOT09-03737440 GGATGTTACTAGGGCACCTTGTTGTCTAAAAGTTGCCACTTGTGATCACACAATTAAGTA[C/T]TAAAATTATGTCAAGTCAACTTTACCGATAATCCGTGGTGAGTATACATCATTGATGTCA

SOT09-06297039 TCTTGATCCACAATTAATAACCCTTTTTTGTTCAAAATATTCTTGTAGTA[T/C]ATATTGTCTAAAATCATTGTAGTTTCGCGATCGAATCTTGAGTATAAAAC

SOT09-07831018 AGCTGAACACATCACAGTTTTCACTTACACATGTAAATAATCAAAAGTTGTTTACCTTAA[C/T]AGAAGAGCAGAGTTGAGGTACGGAATTCTTATGGGAAAGAACACTGGCAGCGATGTTGCG

SOT09-30793211 TGTAAATATGGCGAAGACGAAATGAAGAAAAGCTCTTTGTCACTATTTGACAAACAGTAA[C/G]AAATACGAGTATTATTTACGTCTTAGCTCAGTCGGTAAATTAATTTTAAATTGATGTTTT

SOT09-48069790 ACATGATTCGCCAATTGCAGCACATTCTGACAAGGAATCACCACTGACCACATTTACACC[A/G]TCTTTGGAGTTGAAGGATAACCAGGCAGGAGTCTTTATGTCTTCTTCCTTAAGAAGCTCG

SOT09-57006822 CTGCTTGTGCGATGTTACATCGTTCCTCAATTTCAGGTCCTGTGCGACTGATGACGACAG[A/C]CACACAAAGTATTGCTGCTAAGCTGAGCAGCTCTGGATTGTTGCGGAGTCAGGCTCTTAT

SOT09-59233184 AACAGCAATGTCGATGTCCGAATTGGCAGCATTTCTAAAATCCGGGAACATCAAAGTAAC[T/C]GAATTTGATGCTTTAATCTGTAGCAGTGGGAGCGAAGTGTTTTATCCGGGAACTAGTTCT

SOT09-60562207 GACAAGAAAATGTTATGGGAAAACGGCAGTAATCGGATCTTGAAGCTACCAGAAGGAGGA[G/A]GTTTTGAACTGGTCTGTCAATGGAATGTGACAGATGAGCCTGTGAATTTGTTGCCATTAG

SOT10-01099402 CTCAGCAACGTTAGTTGCTTTAATATGCAAGAGGTAAGGATCACTCAGTTCTCACATTTG[G/T]AAGCTACAGAAAGAATGCATCTGCTTCATATGCTGTTGCTTCTATGTTGATCAAGCTATT

SOT10-04572452 GCGGATATAACTTAGCAAAGATGGATACTTACCGAAGATAACAATTGAGAATCCAATCAC[A/G]AACACACGTTTCAACACATTTCCAACTGCATGTGTAAGAGGTGCCACCCTCTCAAGGGTG

SOT10-48721713 TATAAACATATCTACAGCATGAATAAACTTATTTAAGGTGCACAGAGCAAATACCTGATA[C/G]GAAGGATCAACAAGAACATCCCAAATTGTTGCATTGAAAGCATGATCTGATGTTGCACGA

SOT10-51906416 GTAATTGTGCATTTTTTTGTGGATTAGGTATCATGGTGATTGGAAGAGTGAAACCCAAAC[C/T]GTGGTCTCATTGCTTGCTTTTATGCATTGGTTAGAAACAGGGACTCTTGTTCTGCACAGT

SOT10-54877576 TTAAGTTTCTGTTTACGCTTCTTTGCTGCTTCCTGGACTATGTTTAAGATTTAGTTTTGC[A/T]TGTTGCACAGGACCAAGGTTTGGATGTCATAGCAGAAGGATTGGATACTTTGAAGAACAT

SOT11-00813728 AGTGCCTGATCTAGCCAATATTCATTGTCAGGAAGTGGACGGAGTCAAAGTTCTTCAACT[T/C]GAAACTGCTGCCGGTGCTGCAATTAAGGTATGCCAAACTTAACTCTGTATCTTTAAATAC

SOT11-04666571 GATTTGTACCAATTATATCTTTGCCACTGGTAAAATATTTCCAGAATTCAATTCCCCATG[T/C]ATGGACGAAGAAAGGCTTTTCCTCTCCACCATTGAAGGCTCCATCCTGCTGAAGGCCATC

SOT11-07361035 TTCAGTCAAATCACGCTTTGACATTAGAGATTTTTGGCCTTGGAAGAAGATTTGGCACAA[T/C]TAGTGCCCCTGAAAGTATCTTGTCTCACTTGGTTAGTGGTTAGAGAAGCCTAAACCACTC

SOT11-08822632 AGGAGTAATCAAGCCACCATCTATGGCCACTGCAACAGCAATGTTAATACTACTGTTATA[T/C]GTGAAACTCTTCCCATCTCTACAACTAGAGTTCACAACAGGATGTTTCACCAATGCAAGT

SOT11-39786711 GACGATGTGGAGACGGTGACGGAGGAGGAGGTCGTAGTTTCTGTGAATGGATCCTCCGAG[A/G]GAGTTTTCAACGGGGAGAACTGCACGGTCAGCGATCCAGAGCTCAACGGCTTGGAATGCA

SOT11-40460264 CAGAATTTAGATAACCTTAAATTTTGTTGTGTTCAGGTACCTTTAGTGGCGTCAAACCGC[A/T]TTGGAAAGGAGATAATTGAGACAGAACATGGGAATAGTGAGATAACATTCTACGGCTACT

SOT11-41458020 AGAGGCTGCTTGTTTTCGAATTCATGGAAAATGGCAATCTGAGAGATTGTCTGGATGGGG[A/C]TTCTGGGAGATATCTGGATTGGAGCACACGGGTCGCAGTTGCTTTTGGAGCTGCACGTGG

SOT11-44338305 GTTTAGGTATGGTATCCTTTGTCAAAAACGCTTGCTGAAAAAGATGCTTGGAAATTTGCC[A/G]AGGAAAAAGATTTGGATATTGTTGTGGTGAATCCAGGCACTGTAATGGGCCCTATTATCC

SOT12-00159029 AGTTGTTTCAGGATTCAAGCCTGTTATATTTCGGGCTACATCAATGGGATCAACATTTGC[A/G]AGACTGCAACCACAAAGAAATGTAAATGTAATTTTTCTAACAATTCAGAATGAGAGCATT

SOT12-01882622 GGAGACATTGGGCTGACTGGTGGCAGGATTTATATAAAATTGGGTTGCAAGCTCTGCTGT[C/T]TTCCTCACATAGTTGTGTCGTTTCTCCATCCGGAGACGAGCAAAACGCAAGGCTGATTGT

SOT12-08029918 CATCTTCCAGAAGCTCCTAACAGTCGGAACATGAAACATACTCTGCTGCAGTTGGAGTCT[G/A]CTTTAATGGGGCCAGACGAAGAGGCAATGAAATCCAGCCGTTATCTTGGTGAAAATATGG

SOT12-45971251 GAAACGCCAAAGAAGCCAATACTCTGCTCCTTTCTTTGAAGAAGTCTAGCAGAATTTTCA[G/A]CATAAATAAGTGATTGGAATAAGTGATATATATGATGATGATGAACAAGTGTACCTCTCG

SOT12-53990134 GTGAATGGCGAAAACACATCTCCCTTTTATAAGTTCTTGAAATCAGCAAAGTGGGGATTA[G/C]TTGGGGATAATATTCAGTGGAATTTTGCTAAGTTCCTCGTTGATAAGAATGGCCAAGTTT

SOT12-58220691 GGGTGTTGCGGACTTGGAAATTCGAGTGTACTGTCTAGTTATTGAGTTTTGAAGGTTTTT[G/C]TTTAATTTGGGTGTTTTGATTTTGTGTTGTGTGTTTGTCATGATTTTCTTTGTATTTGGG
